# Lights and Shadows of DMSO as Solvent for Tin Halide Perovskites

**DOI:** 10.1002/chem.202103919

**Published:** 2022-01-05

**Authors:** Jorge Pascual, Diego Di Girolamo, Marion A. Flatken, Mahmoud H. Aldamasy, Guixiang Li, Meng Li, Antonio Abate

**Affiliations:** ^1^ Helmholtz-Zentrum Berlin für Materialien und Energie GmbH Hahn-Meitner-Platz 1 14109 Berlin Germany; ^2^ Department of Chemical Materials and Production Engineering University of Naples Federico II 80125 Naples Italy; ^3^ Egyptian Petroleum Research Institute 11727 Cairo Egypt; ^4^ Key Lab for Special Functional Materials Henan University 475004 Kaifeng China

**Keywords:** lead-free systems, perovskites, perovskite crystallization, perovskite solar cells, tin oxidation

## Abstract

In 2020 dimethyl sulfoxide (DMSO), the ever‐present solvent for tin halide perovskites, was identified as an oxidant for Sn^II^. Nonetheless, alternatives are lacking and few efforts have been devoted to replacing it. To understand this trend it is indispensable to learn the importance of DMSO on the development of tin halide perovskites. Its unique properties have allowed processing compact thin‐films to be integrated into tin perovskite solar cells. Creative approaches for controlling the perovskite crystallization or increasing its stability to oxidation have been developed relying on DMSO‐based inks. However, increasingly sophisticated strategies appear to lead the field to a plateau of power conversion efficiency in the range of 10–15 %. And, while DMSO‐based formulations have performed in encouraging means so far, we should also start considering their potential limitations. In this concept article, we discuss the benefits and limitations of DMSO‐based tin perovskite processing.

## Graphical abstract

**A dead‐end for DMSO**? The crystallization of tin halide perovskites into thin‐films is uncontrolled. The strongly‐binding solvent DMSO and its tolerance to additives helped here. However, device performance stagnates around 10–15 %. The oxidation of tin by DMSO could explain this. Efforts to substitute it are promising, but still lag behind. Addressing solution properties from a fundamental perspective will allow developing DMSO‐free systems.

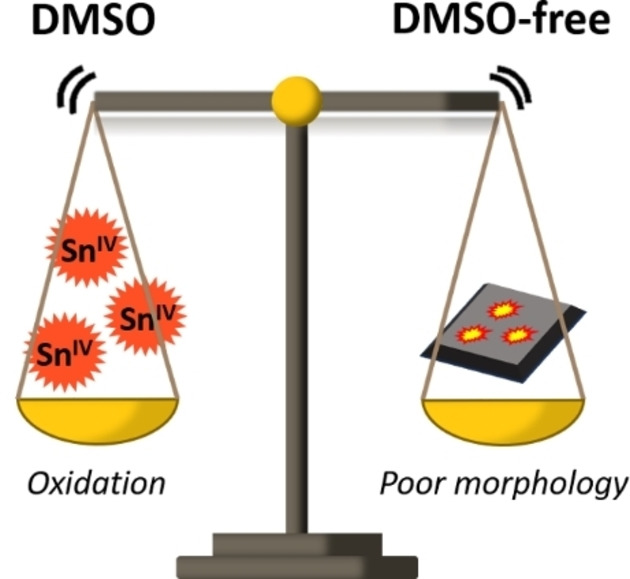

## Introduction

Tin halide perovskites have been successfully implemented in photovoltaic devices during the last decade since their first reports in 2014.[^1^, ^2^] The motivations behind are not scarce, considering their lower toxicity than lead‐based perovskites[^3^, ^4^] and their close‐to‐ideal bandgap.[^5^, ^6^] Nevertheless, the highest reported power conversion efficiencies (PCE) only recently surpassed 14 %,^[7]^ still lagging far behind their theoretical expectations. In addition, the majority of the studies reported in 2021 could only achieve PCE values in the range of 7–10 %, pointing out their low reproducibility. To understand the origin of these limitations, we have to consider the particular chemical nature of tin halide perovskites. Tin salts used for perovskite solar cells (PSCs) are based on Sn in its oxidation state +2, which is not stable to oxygen (air) and can easily be oxidized to +4. Furthermore, solution‐processed tin halide perovskites present a faster crystallization process that is difficult to control compared to lead‐based ones.[^6^, ^8^, ^9^] These two aspects are sources of defects that negatively impact the device performance and enhance the p‐doped character of the material.[^10^, ^11^]

**Figure 1:**
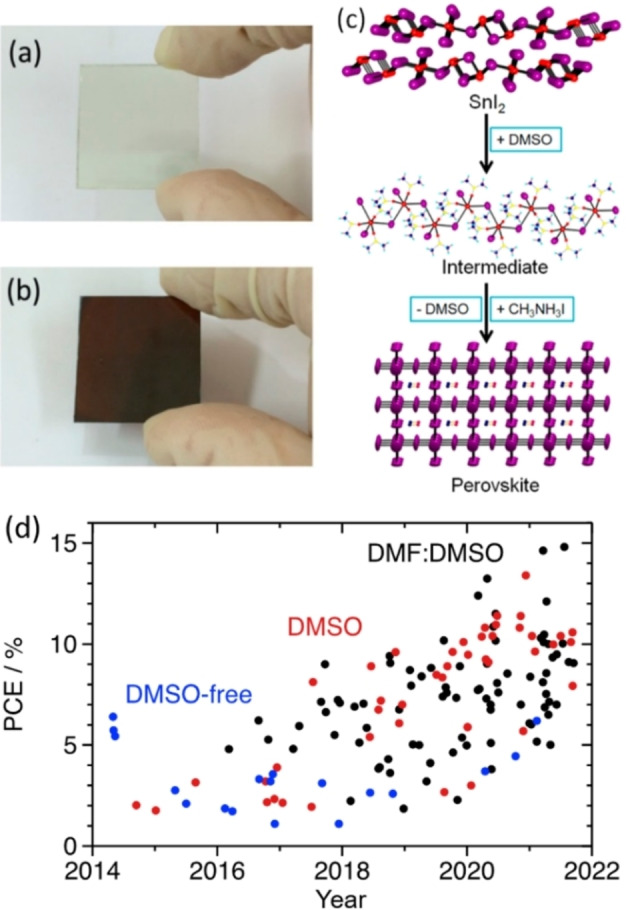
MAPbI_3_ films (a) before and (b) after annealing treatment. Reprinted with permission from Ref. [15]. Copyright 2015 American Chemical Society.^[15]^ (c) MASnI_3_ perovskite film formation from SnI_2_ through the SnI_2_⋅3DMSO intermediate. Reprinted with permission from Ref. [16]. Copyright 2015 American Chemical Society.^[16]^ (d) Highest PCE values for each solution‐processed tin‐based PSCs study reported in the literature.

In this regard, dimethyl sulfoxide (DMSO) as a solvent was a critical factor in developing both lead and tin halide perovskites, owing to its ability to decrease the crystallization rate. Solvents like γ‐butyrolactone (GBL) or *N*,*N*‐dimethylformamide (DMF), the first ones employed to deposit lead halide perovskite for solar cells, lack a solid binding to the metal halide species. DMF presents a 1 : 1 coordination with PbI_2_ and a Pb–O distance of 2.431 Å,^[12]^ whilst DMSO shows a 1 : 2 ratio and a Pb–O distance of 2.386 Å, along with a higher boiling point.^[13]^ This stronger coordination of the DMSO to the metal slows down the perovskite crystallization process through the formation of a methylammonium iodide (MAI)‐PbI_2_‐DMSO intermediate phase before the annealing step (Figure 1a–b),[^14^, ^15^] This effect was easily transferable to its tin counterparts, where the formation of SnI_2_⋅xDMSO intermediate species facilitated a more homogeneous nucleation and slower crystal growth (Figure 1c).^[16]^ This work by Hao et al. paved the way for implementing DMSO as the critical component in tin halide precursor solutions, and DMSO‐based formulations (i.e. pure DMSO, DMF:DMSO) quickly substituted pure DMF as the perovskite precursors solvent (Figure 1d). Now, it represents the standard conditions.

Extensive additive engineering in DMSO‐based perovskite precursor solutions enabled substantial progress, mainly relying on SnF_2_[^17^, ^18^] and bulky cations leading to low dimensional perovskites[^19^, ^20^, ^21^] or a varied selection of antioxidants.[^22^, ^23^, ^24^, ^25^] Notably, solar cells based on additive‐free tin perovskites practically do not work.^[18]^ In contrast, the highest efficiencies from tin PSCs are commonly achieved by adopting complex multi‐additive strategies.[^7^, ^26^] With well‐established protocols and after testing many additives and treatments, the field seems to be clogging around 15 % PCE (Figure 1d). This situation markedly differs from lead‐based perovskite photovoltaics, where reproducible efficiencies above 20 % are obtained even with pristine MAPbI_3_.^[27]^

In this contribution we analyze the role of DMSO in the current state of tin PSCs, focusing on its oxidative behavior and on alternative solutions and their limitation. We aim to advance the efforts to adopt inert solvents to process of tin perovskites, which we believe is an important step to close the gap to lead‐based perovskites.

### DMSO as Sn^II^ Oxidant

Although the oxidative activity of DMSO is well‐known, it took almost a decade to observe the direct oxidation of Sn^II^ to Sn^IV^ from DMSO in perovskite precursor inks. The dipolar aprotic properties of DMSO arise from the sulfoxide bond, prone to negative charge delocalization on the oxygen atom. The excess negative charge density on the oxygen atom can coordinate cations or positively charged molecular moieties, allowing ionic compounds such as tin halides to dissolve. A positive charge density is localized on the sulfur atom, yet less exposed to the surroundings due to steric hindrance from the methyl groups. Notably, in the presence of suitable reactants, the dipolar sulfoxide bond can break with the release of oxygen and the formation of dimethyl sulfide in the formal reduction of DMSO. In synthetic organic chemistry DMSO is a traditional mild oxidant, with the Swern^[28]^ and the Moffatt^[29]^ reactions being the two most representative examples. Remarkably, in the presence of halides the DMSO oxidant behavior can be empowered,[^30^, ^31^, ^32^] and efficient oxidation of iodide to iodine has been reported and exploited to design metal‐free oxidants.^[33]^

**Figure 2:**
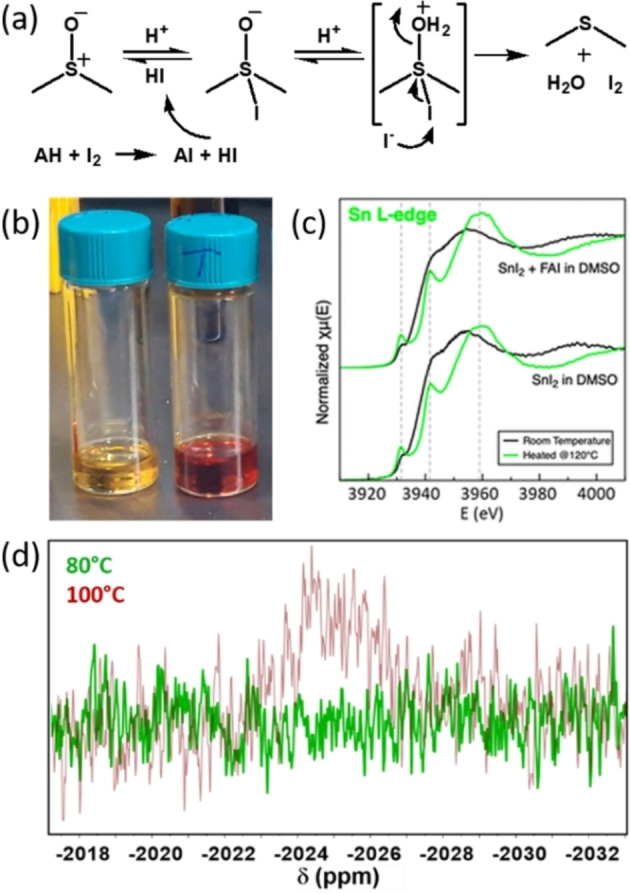
(a) Reaction mechanism of DMSO reduction in the presence of iodide and protons (hydroiodic acid here). The reaction proceeds through the complexation of H^+^ by the negatively charged oxygen and iodide to the positively charge sulfur atom of DMSO. The oxygen is lost as water and the iodide as iodine. Formally, iodide is the reductant and DMSO is the oxidant. Sn^II^ present in the solution could then be oxidized by I_2_.^[33]^ (b) The color change of the DMSO‐based tin perovskite precursor solution from yellow (containing only Sn^II^) to red (also having Sn^IV^) upon heating at 100 °C in inert atmosphere. Reproduced from Ref. [35] with permission from the Royal Society of Chemistry.^[35]^ (c) XANES characterization at the Sn L‐edge highlighting the appearance of Sn^IV^ after heating at 120 °C the DMSO‐based SnI_2_ and tin perovskite solutions. Reprinted with permission from Ref. [34]. Copyright 2020 American Chemical Society.^[34]^ (d) ^119^Sn NMR of DMSO‐based perovskite solution after heating at 80 °C or 100 °C for 0.5 h. The signal at a chemical shift attributable to Sn^IV^‐iodide complex is observed only after heating at 100 °C within this time span. Reproduced from Ref. [35] with permission from the Royal Society of Chemistry.^[35]^

Along with the change in color from yellow to dark red of the tin iodide perovskite solution, the formation of Sn^IV^ in a DMSO‐containing ink has been confirmed with X‐ray absorption near‐edge spectroscopy (XANES)^[34]^ and ^119^Sn NMR^[35]^ (Figure 2). The group of Hayase showed an increase in the Sn^IV^ signal by X‐ray photoelectron spectroscopy (XPS) upon ageing a CsSnI_3_ perovskite solution and a decrease of the Seebeck coefficient in thermoelectric devices.^[36]^ This observation suggests that the oxidation occurs even at room temperature, yet with slow dynamics and with Sn^IV^ hardly detectable. For our NMR measurements, when heating the perovskite solution at 80 °C for 3 h, we could not observe the Sn^IV^ signal that we saw at 100 °C. Unfortunately, the nuclei of study and measurement conditions determine the limit of detection (LOD) of NMR, which is too high and therefore unsuitable for characterizing the ppm/ppb concentration level required to assess the quality of semiconductors. Speculating that every Sn^IV^ ion in the precursor solution enters into the perovskite lattice creating a trap state, to maintain the trap density in the perovskite film within the 10^14^–10^15^ cm^−3^ range (common trap density in lead halide perovskite films), the concentration of Sn^IV^ has to be lower than 10^−8^ M (with 1 M Sn^II^ and a Sn^II^ concentration of 10^23^ atom/cm^3^ in the perovskite lattice). Therefore, even if the solution is not red and NMR does not detect Sn^IV^, this can still be included in excessive amount in the perovskite. The results on thermoelectrics from the group of Hayase along with vast literature, where sizeable amounts of Sn^IV^ are detected in the film surface by XPS, support our considerations.

### Alternatives to DMSO

The increase in PCE up to 15 % demonstrates that additive engineering allows the control of the doping density of tin perovskites even from DMSO‐inks to a certain extent. However, removing this reagent would be a much more drastic and resolutive method. Previous reports not employing DMSO in solution‐processed tin perovskite fabrication are mainly limited to pure DMF (Figure 1d). Unfortunately, this solvent has a lower binding ability to the metallic center than DMSO. Therefore, the crystallization rate increases, which leads to poor thin‐film morphology.^[16]^ It is worth mentioning though the possibility to produce highly purified tin compounds through the complexation of Sn^II^ to DMF.^[37]^

**Figure 3:**
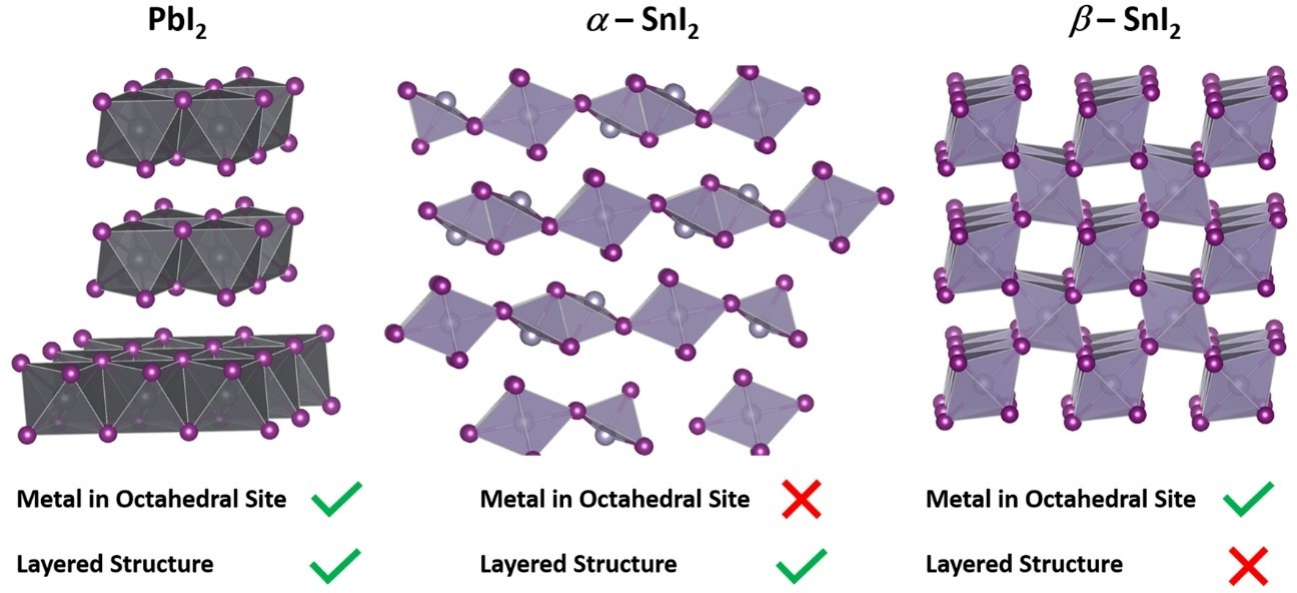
Crystal structures of PbI_2_ and SnI_2_ precursors.

A possible route to remove DMSO is the 2‐step deposition or sequential deposition, consisting of the deposition of SnI_2_ and the subsequent conversion to perovskite. In the case of lead perovskites, PbI_2_ is usually deposited from a DMF solution in the first step, and the same could apply to tin perovskites. However, the conversion of SnI_2_ to perovskite is less straightforward than for PbI_2_ ^[38]^ and the reason might stem from the different crystal structures of iodide precursors (Figure 3). PbI_2_ has a layered structure with sheets of edge‐sharing lead‐iodide octahedra, which promotes the diffusion of the AX precursor to form the perovskite lattice. SnI_2_, on the other hand, has two different crystal structures. The most stable at room temperature is the *α*‐SnI_2_ (monoclinic), which is layered but with 2/3 of tin atoms involved in non‐octahedral coordination with halides.^[39]^ The structure is stable at slightly higher temperatures (and in evaporated thin films) as *β*‐SnI_2_ (hexagonal) is a tridimensional network of edge‐sharing tin‐iodide octahedra.^[40]^ Both phases are structurally different from PbI_2_ and careful engineering of the AX solution is required to enhance the degree of conversion to perovskite, suggesting that SnI_2_ is a perovskite precursor of lower quality than PbI_2_. Notably, Sn_1‐x_Pb_x_I_2_ compounds crystallize in the PbI_2_ structure,^[39]^ which could explain the relatively more effective processing of mixed lead‐tin perovskites in respect to pure tin.

**Figure 4:**
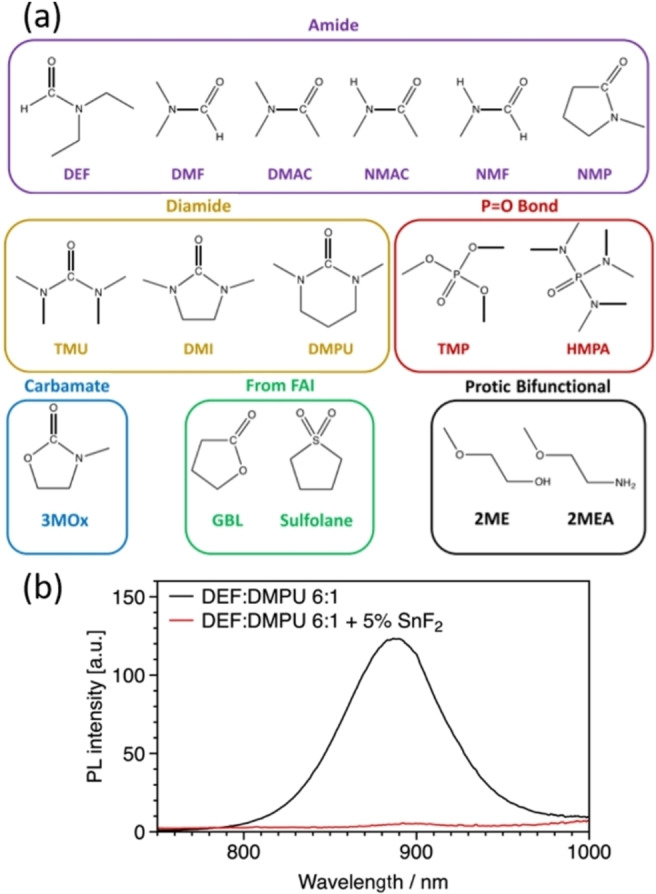
(a) The list of solvent alternatives to DMSO can form a >1 M FASnI_3_ solution. Adapted with permission from Ref. [41]. Copyright 2021 American Chemical Society.^[41]^ (b) PL of FASnI_3_ thin films processed from DEF:DMPU solvent mixture with and without SnF_2_.

Considering the 1‐step antisolvent‐based deposition route, we reported 16 solvents suitable for replacing DMSO (or other sulfoxides, all of them oxidizing Sn^II^) and guidelines for further identification (Figure 4a).^[41]^ Through the combination of *N*,*N*‐diethylformamide (DEF) and *N*,*N*′‐dimethylpropyleneurea (DMPU), we demonstrated solar cells outperforming those based on DMSO, proving that the approach is viable. Moreover, recent data from our laboratory indicate that the films processed from DMSO‐free precursors have a more defined intrinsic character than self p‐doped DMSO‐based films, even in the presence of SnF_2_.^[42]^

The most relevant challenge is the control of the morphology and thickness of tin perovskite thin films. In our investigation with DEF:DMPU mixtures, the window for the antisolvent dripping is very narrow, allowing a tolerance of 1–2 seconds. This behavior gives small room for optimization. Additionally, the antisolvent appears to interact with the wet film beyond the simple solvent washing, which induces perovskite precipitation and tends to wash the film thoroughly. When the precipitation occurs, it is very fast and the film immediately turns black. This process results in small grain size morphology and difficulties in obtaining a specular film. At the same time, it is more common to get a greyish film due to the high roughness, either at the surface or the interface with the substrate. The 14.6 % record tin PSCs are based on a 200 nm thick perovskite film,^[7]^ suggesting that controlling the thickness is also challenging from DMSO, but this exacerbates in DMSO‐free systems.

The Donor Number (D_N_) is an important parameter that describes the ability to coordinate a positively charged species, such as Sn^II^. The high DMSO D_N_ of 29.8 kcal/mol might explain the ability of this solvent in retarding the perovskite crystallization: the halide must compete with a solid binding solvent for the first coordination shell of Sn^II^ and this results in a kinetic barrier for the perovskite crystallization, slowing down the process and allowing to control the morphology of the film. DMF, for instance, having a lower D_N_ (26.6 kcal/mol) than DMSO, is less effective in the competition with the halide for the Sn^II^ coordination, resulting in a faster crystallization. Interestingly, if we consider DEF and DMPU, which compose the solvent system we identified as potentially replacive of DMSO, both of these solvents have a D_N_ slightly larger than DMSO, supporting the consideration that a large D_N_ is required to obtain a high‐quality perovskite film. However, this picture might be too simplistic. In fact, along with descriptors for the solvent strength, the work by Jiang et al. disclosed that the path through which the precursor complex is formed is critical.^[7]^ The formation of Sn−I‐DMSO complex in situ, starting from I_2_ and metallic Sn, leads to a perovskite film of higher quality. Sekimoto and coworkers proposed that A_N_ (the Acceptor Number, a measure for the Lewis acid strength) might also be relevant for the tin perovskite crystallization.^[43]^ They could obtain the inverse temperature crystallization of FASnI_3_ only from *γ*‐valerolactone, which has a low A_N_ (13.6) and thus weakly coordinate iodide. DMSO has a relatively large A_N_ of 19.3, compared to the value of 16.0 for DMF, thus reducing the iodide activity. The solvents based on amide or diamide functional groups, such as DEF and DMPU, have a lower A_N_ than DMSO and, therefore, weaker coordination with the iodide, which increases the crystallization rate. Another challenge in replacing DMSO is the difficulty in adopting common doping strategies, such as SnF_2_. If one introduces SnF_2_ into the DEF:DMPU tin perovskite precursors ink the photoluminescence (PL) is completely quenched (Figure 4b). This behavior is totally unexpected since SnF_2_ is reproducibly reported to reduce the background carrier density of tin perovskite, resulting in an intense PL. A possible reason for that might be the low solubility of SnF_2_ in DEF:DMPU. This observation illustrates the poor or problematic transferability of doping strategies from DMSO‐based to DMSO‐free routes.

### Understanding and Manipulating Tin Perovskite Crystallization

The approaches for discovering DMSO‐free perovskite fabrication processes highlight the difficulty to derive good polycrystalline tin‐based perovskite thin films.^[41]^ Previous studies confirm that controlled crystallization is the key to reaching a good surface coverage.[^44^, ^45^] This correlation applies to lead‐free perovskites as well as to their lead containing counterparts. The latter in particular has already been extensively investigated.[^44^, ^46^, ^47^, ^48^] However, the topic of crystallization from a colloidal solution is still not fully understood since new approaches of the non‐classical nucleation in combination with classical nucleation theory open up a variety of possible nucleation and growth pathways that are difficult to attribute precisely to the perovskite film crystallization.[^49^, ^50^]

Hence, there is a need to investigate further and understand the physicochemical behavior of tin‐based precursor solutions to uncover the crucial property actually enabling a perovskite film formation using DMSO, which is at first sight not present when using other solvents with similar characteristics. First attempts in providing nucleation pathways for better control of the crystallization kinetics are summarized in a review by Wang et al.,^[45]^ addressing the classical and non‐classical nucleation and growth theories. According to the classical nucleation theory (CNT), nucleation is a simple thermodynamic model, including the creation of a solid particle (nuclei) in a liquid medium. LaMer and Dinegar summarized this first nucleation approach from CNT including a growth process as a function of time.^[51]^ However, due to today's advanced (in situ) measurements, researchers uncover that the CNT is not sufficient to cover the observed processes. Different experimental pathways of nucleation and growth become possible, covered with the broad term of non‐classical nucleation theory.

**Figure 5:**
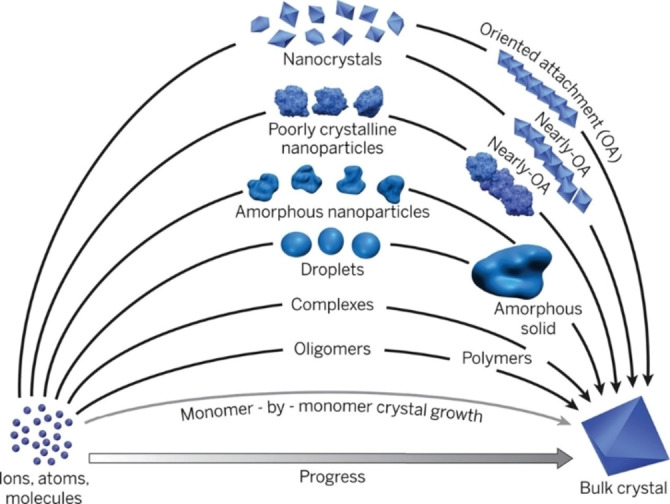
Pathways to crystallization by particle attachment. In contrast to monomer‐by‐monomer addition as envisioned in classical models of crystal growth (grey curve). From Ref. [50]. Reprinted with permission from AAAS.^[50]^

Considering the complexity of nucleation and growth pathways presented by De Yoreo et al. (Figure 5) following the non‐classical nucleation theory, a better understanding of the precursor solution is essential for further developing tin‐based solar cells. Methodologies like small‐angle X‐ray scattering (SAXS) can reveal insights into the physicochemical properties of the precursor solution depending on the solvent.^[52]^ Confirming that exchanging DMSO with DMF has a direct and significant effect on the scattering behavior of the solution and is additionally changing the additive‘s behavior, for example SnF_2_.^[18]^ In‐situ grazing incidence wide‐angle scattering (GIWAXS) proofs itself to be another valuable technique to investigate structural details during the crystallization and growth process of the perovskite thin film.^[21]^ However, this methodology is challenging to apply for tin‐based perovskites due to their atmospheric instability. Therefore, special conditions concerning the sample environment would have to be provided.

## Summary and Outlook

DMSO has been a critical component in PSC research for processing high quality, compact perovskite thin films. In particular, its ability to strongly coordinate the metallic centers allowed the control of the too fast crystallization rate in tin halide perovskites. However, solutions based on this solvent require many additives to process functional thin films, and the reported efficiency values start to clog up in the 10–15 % range. We believe the recently described oxidation of Sn^II^ by DMSO holds a big responsibility for it, suggesting the presence of intrinsic limitations for this so‐optimized system. Highly employed additives like SnF_2_ may have a role in minimizing the damages, but only fully removing DMSO would successfully address the fundamental problem. Attempts to find suitable alternatives have not been decisively successful yet, pointing out the complexity of the crystallization process. Therefore, it is now the time to step back, observe the field from a different perspective, and invest new efforts in understanding the fundamentals of these materials, to give the correct answer to it. Studying the characteristics of the nucleation and crystal growth processes in tin halide perovskites would allow a more robust control on thin‐film fabrication, opening the door to DMSO‐free alternatives and yielding layers of higher quality on a wide range of substrates.

## Conflict of interest

The authors declare no conflict of interest.

1

## Data Availability

The data that support the findings of this study are available from the corresponding author upon reasonable request.
